# *Sutherlandia frutescens* may exacerbate HIV-associated neuroinflammation

**DOI:** 10.1186/s12952-015-0031-y

**Published:** 2015-07-18

**Authors:** Luan Dane Africa, Carine Smith

**Affiliations:** Department of Physiological Sciences, Stellenbosch University, Private Bag X1, Matieland, 7602 South Africa

## Abstract

**Background:**

Neuroinflammation is central to the aetiology of HIV-associated neurocognitive disorders (HAND) that are prevalent in late stage AIDS. Anti-retroviral (ARV) treatments are rolled out relatively late in the context of neuroinflammatory changes, so that their usefulness in directly preventing HAND is probably limited. It is common practice for HIV+ individuals in developing countries to make use of traditional medicines. One such medicine is *Sutherlandia frutescens* - commonly consumed as a water infusion. Here its efficacy as an anti-inflammatory modality in this context was investigated in an in vitro co-culture model of the blood–brain barrier (BBB).

**Methods:**

Single cultures of human astrocytes (HA), HUVECs and primary human monocytes, as well as co-cultures (BBB), were stimulated with HIV-1 subtype B & C Tat protein and/or HL2/3 cell secretory proteins after pre-treatment with *S.frutescens* extract*.* Effects of this pre-treatment on pro-inflammatory cytokine secretion and monocyte migration across the BBB were assessed.

**Results:**

In accordance with others, B Tat was more pro-inflammatory than C Tat, validating our model. *S.frutescens* decreased IL-1β secretion significantly (*P* < 0.0001), but exacerbated both monocyte chemoattractant protein-1 (*P* < 0001) – a major role player in HIV-associated neuroinflammation – and CD14+ monocyte infiltration across the BBB (*P* < 0.01).

**Conclusions:**

Current data illustrates that the combined use of HL2/3 cells and the simulated BBB presents an accurate, physiologically relevant in vitro model with which to study neuroinflammation in the context of HIV/AIDS. In addition, our results caution against the use of *S.frutescens* as anti-inflammatory modality at any stage post-HIV infection.

**Electronic supplementary material:**

The online version of this article (doi:10.1186/s12952-015-0031-y) contains supplementary material, which is available to authorized users.

## Introduction

Neuroinflammation in the context of HIV/AIDS is known to have onset soon after infection with the virus and is associated with the HIV-associated neurocognitive disorders (HAND) that are prevalent in late stage AIDS [1]. Conventional anti-retroviral treatments are rolled out relatively late in the context of neuroinflammatory changes, so that their usefulness in directly preventing HAND is probably limited. A recent multi-centre study in more than 800 HIV+ patients [2], reported that high rates of neurocognitive impairment persist at all stages of HIV infection, despite modern anti-retroviral treatment and immune reconstitution regimes. Furthermore, in the same study, neurocognitive impairment was consistently associated with lowest CD4 counts. From this, it is clear that early prevention strategies to limit the extent of neuroinflammation, is required to positively influence the longer-term prognosis in terms of not only HAND, but also disease progression.

Traditional medicine is commonly used by those living with HIV infection, particularly in developing countries. One such complementary medicine that is widely used, is *Sutherlandia frutescens (Fabaceae alt. Leguminosae, Goldblatt & Manning 1812) –* a herb that is commonly consumed in the form of a tea. Several beneficial effects relevant to HIV/AIDS have been reported for this herbal remedy. Firstly, it was shown to directly inhibit activity of HIV-target enzymes [3]. Secondly, the use of *S. frutescens* has been associated with benefits in the peripheral compartment - decreased psychological stress levels [4, 5] and preservation of skeletal muscle mass [6] suggests a less catabolic state and maintenance of overall body strength. Thirdly, central uptake and effect was reported in separate studies reporting anxiolytic [7] and anti-convulsant [8] effects of *S. frutescens* via modulation of GABAergic neurotransmission.

As a result of these promising data, the use of *S. frutescens* in the context of HIV/AIDS is currently endorsed by the Ministries of Health of several African nations [9]. Even though *S. frutescens* was recently implicated in herb-drug interactions which may lead to therapeutic failure and/or increased drug toxicity in the context of HIV anti-retrovirals specifically [10–13] clinic staff continue to recommend its use to HIV+ patients still waiting for ARV roll out, for management of secondary symptoms of HIV/AIDS in otherwise untreated patients (personal communications to CS).

From the literature reviewed, *S frutescens* is known to be absorbed and have central activity, but its potential role in modulation of neuroinflammation has not been assessed. This fact, together with the fact that it is currently being used widely in the time frame of disease progression where treatments for neuroinflammation should be applied, warrants investigation into the potential of *S. frutescens* as anti-inflammatory modality in the context of HIV-associated neuroinflammation. Therefore, the effect of *S. frutescens* treatment on monocyte migration across a simulated blood–brain barrier was assessed in vitro. In addition, effects on the secretion of inflammatory modulators by various cell types were investigated.

## Materials and methods

### Cell culture

Single cultures of primary human cerebrocortical astrocytes (HA) (Sciencell, USA) and Human Umbilical Vein Endothelial Cells (HUVECs) (Lonza, Germany) were maintained at 37 °C in a humidified 5 % CO_2_ in high glucose DMEM (Life Technologies Corp., USA) supplemented with 10 % FCS (Biochrom, Germany) and 1 % N2 Supplement (Life Technologies Corp., USA) and complete EGM (Lonza, Germany) respectfully. HL2/3 cells (obtained through the NIH AIDS Reagent Program, Division of AIDS, NIAID, NIH: HL2/3 from Dr. Barbara K. Felber and Dr. George N. Pavlakis), HeLa derived cells producing high levels of Gag, Env, Tat, Rev and Nef proteins, were maintained at the conditions mentioned above in high glucose DMEM (Life Technologies Corp., USA) supplemented with 10 % FCS (Biochrom, Germany). Cells were routinely sub-cultured before reaching confluence.

Cell numbers were determined using a haemocytometer following trypsinization and trypan blue staining. For 3-[4,5- dimethylthiazol-2-yl]-2,5-diphenyl tetrazolium bromide (MTT) assays, HA and HUVECs were seeded in 6 well cell culture plates (500000 cells/well). For all other single culture experiments all cell types were seeded in 6 well cell culture dishes at the aforementioned cell density.

In order to simulate the blood–brain barrier, co-cultures of HA and HUVECs were established on opposite sides of fibronectin (BD Biosciences, USA) coated 3 μm pore size tissue culture inserts (BD Biosciences, USA) [14].

All cell culture experiments were done in triplicate and repeated a minimum of three times.

### Preparation of *S. frutescens* aqueous extract

Commercially available plant material was kindly donated by Mr Ulrich Feiter (Parceval Pharmaceuticals Pty Ltd). *Sutherlandia frutescens* plants were cultivated from commercial stock seed which have been previously taxonomically verified as *S. frutescens var. SU1 (registered product code 02P0058)*, harvested (well after flowering and seeding phase) and dry milled (leaves and stems only) by Parceval Pharmaceuticals Pty Ltd (Wellington, South Africa) using proprietary procedures. A warm water extract of dry-milled *S. frutescens* (moisture content 16.41 %) was prepared in boiling distilled water (25 mg/ml) using methods previously described for in vivo treatment [4, 7] and then sterile filtered using filter pore size 0.22 μm.

### *S. frutescens* dose response cell viability assay

In order to determine the highest dose of *S. frutescens* tolerated with the least amount of cell death, human astrocytes, HUVECs and primary human monocytes were incubated with 50, 500 and 5000 μg/ml *S. frutescens* extract for 24 h.

Cell viability was assessed using a modified version of the MTT assay described by Gomez and colleagues [15]. The assay is based upon the principle of reduction of MTT into blue formazan pigments by viable mitochondria in healthy cells. At the end of the experiment, the medium was removed from the 6 well plates and the cells washed twice with PBS. MTT (0.01 g/ml) was dissolved in PBS, and 500 μl was added to each well dish. Cells were subsequently incubated for 1 h at 37 °C in an atmosphere of 5 % CO_2_. After the incubation period, cells were washed twice with PBS, and one ml of HCl–isopropanol–Triton (1 % HCl in isopropanol; 0.1 % Triton X- 100; 50:1) was added to each well and gently agitated for 5 min. This lysed the cell membranes and liberated the formazan pigments. The suspension was then centrifuged at 131 × g for 2 min. The optical density (OD) was determined spectrophotometrically at a wavelength of 540 nm and the values expressed as percentages of control.

### Full length HIV-1 subtype B & C Tat protein stimulation

Full length synthetic Tat proteins were kindly provided by Professor Ranga Udaykumar of the Jawaharlal Nehru Centre for Advanced Scientific Research (Bangalore, India) and were synthesized and purified as previously described [16]. Tat proteins were reconstituted and subsequently diluted in Tris-Cl buffer (20 mM, pH8) supplemented with 1 mM DTT.

Human astrocytes, HUVECs, primary human monocytes as well as simulated BBB co-cultures were stimulated with either protein (10 ng/ml) for 2.5 h and 24 h, after which culture media was collected and stored at −80 °C for subsequent analyses. In order to test the efficacy of *S. frutescens* as a modulator of neuroinflammatory processes, cells were pre-treated for 4 h and 24 h respectively prior to HIV-1 Tat protein stimulation. Following stimulation culture supernatants were collected and stored at −80 °C until further analysis.

### HL2/3 Cells – A more representative in vitro model of HIV-1 infection

As previously mentioned, HL2/3 cells produce and secrete high levels of most HIV-1 subtype B proteins into their culture media, and for this reason it was decided to co-culture these cells with the simulated BBB cultures in order to mimic the neuroinflammatory milieu at the interface between the infected central nervous system (represented by the HL2/3 cells seeded into the wells of a 24 well culture plate into which the tissue culture inserts, on which the simulated BBB has been constructed, are placed) and the neurovasculature (i.e., the BBB represented by the in vitro simulated BBB cultures). Firstly, to assess the effect of the HL2/3 derived HIV-1 proteins on the individual cell types used to construct the in vitro BBB, HL2/3 cells were seeded into 6 well plates at 200 000 cells per well and allowed to adhere to the culture surface. Once the HL2/3 cells had adhered, culture media was replaced. HL2/3 conditioned media was collected at 2.5 h and 24 h from separate cultures, and this media was then used to stimulate human astrocytes, HUVECs and primary human monocytes for either 2.5 h or 24 h. Also, as in the aforementioned section outlining the HIV-1 Tat experiments, cells were pre-treated with *S. frutescens* for either 4 h or 24 h prior to stimulation. Culture supernatants were collected post stimulation and stored at −80 °C until further analysis.

The aforementioned experiment was repeated in the co-culture system, with the omission of the 24 h time point. HL2/3 cells were seeded into 24 well plates at 50 000 cells/well and allowed to adhere. Culture media was refreshed after which the BBB co-cultures were transferred to the wells containing the HL2/3 cells. BBB co-cultures were exposed to the HL2/3 cells for a period of 2.5 h, after which culture supernatants were collected and stored at −80 °C for further downstream analyses. Additional BBB co-cultures were treated with *S. frutescens* for 4 h prior to stimulation.

### Pro-inflammatory cytokine & chemokine analysis

Monocyte chemoattractant protein-1 (MCP-1), a key role player in HIV-1-associated neuroinflammation, was measured in all supernatants by a conventional ELISA kit (Biolegend, San Diego, CA), used according to the manufacturer’s instructions.

IL-1β was measured in all co-culture supernatants by AlphaLISA (PerkinElmer, Waltham, MA), according to the manufacturer’s instructions.

### Monocyte/macrophage transmigration

Transmigration of both infected and uninfected inflammatory cells, in particular those from the monocyte macrophage lineage, play a major role in the aetiology of HIV-1-associated neuroinflammation. For this reason monocyte transmigration was assessed in the BBB co-cultures by adding primary human monocytes to the top of the insert, allowing the cells to migrate in response to the various stimuli for 2.5 h, after which the BBB inserts and cells in the bottom of the well were fixed in 4 % paraformaldehyde and stained with a FITC-anti-human CD14 antibody (Biolegend, San Diego, CA). CD14 is a specific marker of cells from the monocyte/macrophage lineage. All CD14+ monocytes on top of the entire insert (unmigrated) and on the bottom of the culture well (migrated) were counted using a fluorescent microscope (Leica, Germany). Cells in suspension were not quantified, since we have previously shown that cell counts in these compartments are independent of interventions/treatments [17].

### Statistical analysis

All statistical analyses were performed using Graphpad Prism Version 5 software (Graphpad Software, La Jolla, CA, USA). Results are expressed as mean ± SD. One- or two- way analysis of variance (ANOVA) as relevant, followed by a Bonferroni *post hoc* test, was used to assess differences between experimental groups and/or time points. Differences were considered to be of statistical significance when *P* value ≤ 0.05.

## Results

Due to the fact that *S. frutescens* had not, up until this point, been tested on the cell types used in this study, it was important to first establish the optimal dose for use in vitro, prior to assessment of *S. frutescens* as an effective anti-inflammatory modality. We defined this optimal experimental dose as the highest dosage which does not result in a significant reduction in cell viability, represented by % MTT reductive capacity, Across all three cell types, a marked, statistically significant reduction in % MTT reductive capacity was observed in the group treated with 5000 μg/ml *S. frutescens* at all time points (*P* < 0.0001 when compared to control; refer to Additional file 1 for graphical representation of data). No statistically significant changes in cell viability were observed for any of the other treatment doses at any time point, and thus the highest of these - 500 μg/ml - was selected as the optimal experimental dose.

MCP-1 responses were evaluated in human astrocytes, HUVEC, primary human monocyte and BBB co-cultures following stimulation with HIV-1 subtype B & C Tat protein, and also HL2/3 conditioned media in the case of astrocytes, HUVECs and monocytes, and co-culturing BBB cultures with HL2/3 cells. The potential of *S. frutescens* as a modulator of inflammation was evaluated by pre-treating cells with *S. frutescens* prior to introduction of the inflammatory stimulus.

**Fig. 1:**
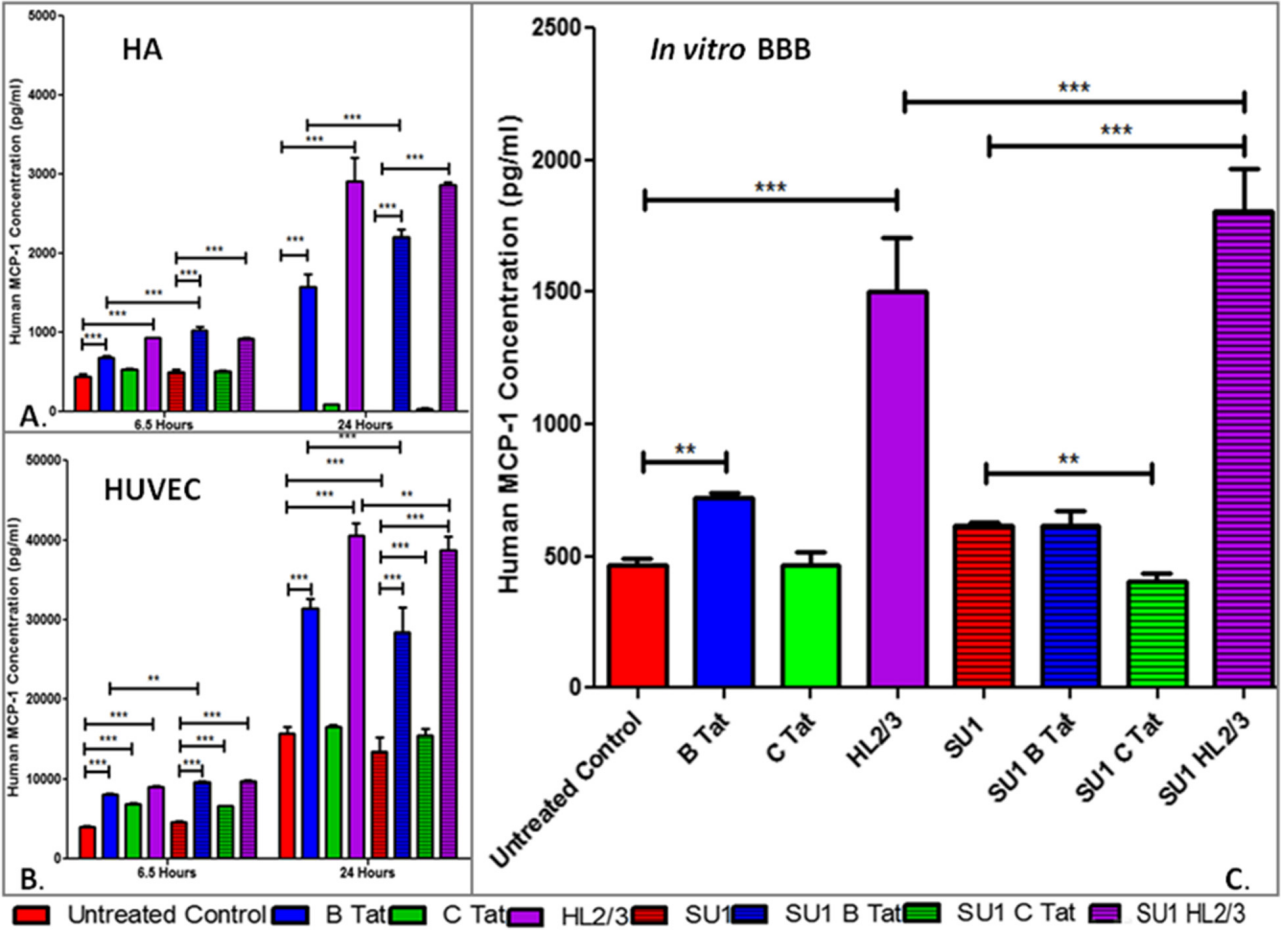
MCP-1 response to HIV-1 proteins in human astrocytes (**a**), HUVECs (**b**) and BBB co-cultures (**c**), with or without pre-treatment with *S.frutescens* extract. SU1 indicates groups pre-treated with *S. frutescens.* SU 1 pre-treatment duration was 4 h for the 6,5 h experiments, and 24 h in the case of the 24 h experiments. Results are expressed as mean ± SD. *** = *P* < 0.0001; ** = *P* < 0.001

In the absence of HIV-associated proteins, control astrocytes secreted low basal levels of MCP-1 at 6.5 h, but no MCP-1 was detectable after 24 h in culture (Fig. 1a). After exposure to either B Tat or HL2/3 cell products, MCP-1 secretion increased significantly over time up to the 24 h point (all *P* < 0.0001 when compared to control). As expected, C Tat elicited no MCP-1 response. Pre-treatment with *S. frutescens* in absence of HIV proteins seemed to reduce basal secretion of MCP-1. However, it exacerbated the response to both B Tat and HL2/3 cell products in stimulated cells at both 6.5 and 24 h (all *P* < 0.0001 when compared to control).

In HUVECs (Fig. 1b), basal MCP-1 secretion followed a similar pattern to that seen in astrocytes at 6.5 h, with the exception of C Tat, which also elicited a basal response in this cell type (*P* < 0.0001 when compared to control). In contrast to astrocytes, this basal secretion was maintained and even relatively enhanced at 24 h. In the absence of HIV proteins, HUVECs responded similarly to astrocytes when *S. frutescens* pre-treated, showing smaller increases in basal MCP-1 levels. In the presence of all HIV protein stimuli employed, MCP-1 secretion increased continuously up to 24 h. Although *S. frutescens* did not exacerbate the response in this cell type, the *S. frutescens-*associated inhibition of the MCP-1 response seen in non-HIV conditions, was insufficient to restore the response after exposure to HIV proteins at 24 h to control levels.

When repeating the intervention protocols in a co-culture simulation of the BBB (consisting of astrocytes, HUVECs and monocytes, Fig. 1c), the net effect of *S. frutescens* that may be expected in an in vivo situation becomes more evident. Pre-treatment with *S. frutescens* had no beneficial effect on the HIV Tat (both B and C) associated MCP-1 response and exacerbated the HL2/3-induced response (*P* < 0.0001). (Monocyte cultures did not secrete detectable levels of MCP-1 under any of the experimental conditions, so most probably did not contribute significantly to this outcome.) These results suggested that HIV Tat proteins are not a therapeutic target of *S. frutescens*. Also, treatment with HL2/3 conditioned media or co-culture with these cells (which contain Tat as well as other HIV proteins) resulted in the most pronounced inflammatory response. Therefore, HL2/3 cells were selected as pro-inflammatory stimulus for all further experiments. Additionally, the fact that these cells secrete a greater repertoire of HIV-1 proteins makes them a more physiologically representative model of infection.

**Fig. 2:**
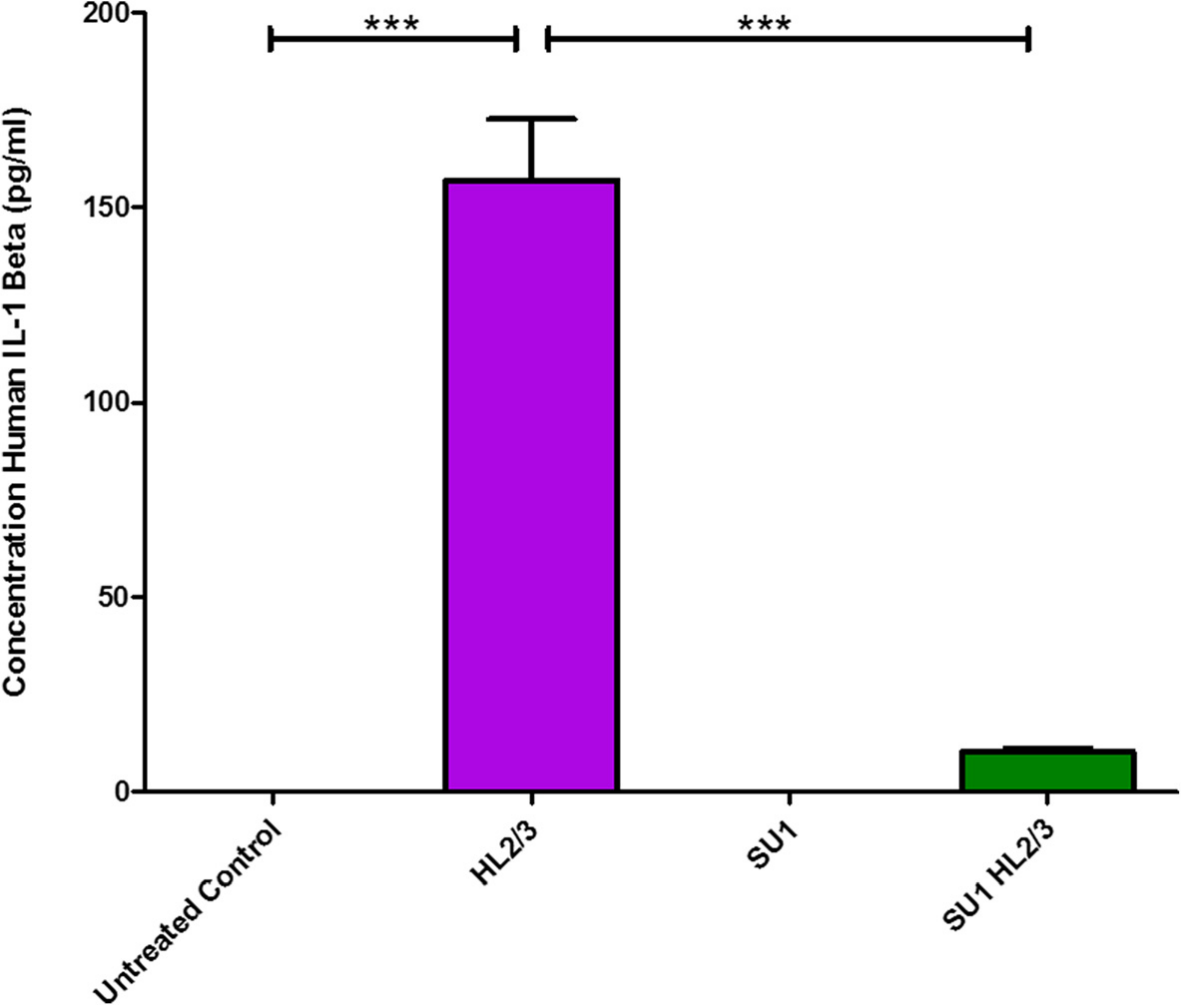
IL-1β response to co-culture exposure of BBB co-cultures to HL2/3 cells, in the presence or absence of *S.frutescens* extract. SU1 indicates groups pre-treated with *S. frutescens* for 4 h*.* Results are expressed as mean ± SD. *** = *P* < 0.0001; ** = *P* < 0.001

IL-1β levels were non-detectable in single cultures of HL2/3 cells (data not shown), so that any IL-1β detected originated from the BBB. IL-1β secretion was evaluated in BBB cultures stimulated by co-culture with HL2/3 cells. Pre-treatment of the BBB cultures with *S. frutescens* was able to effectively inhibit the IL-1β response following co-culture with HL2/3 cells, so that the response was similar to basal secretion levels (Fig. 2).

**Fig. 3:**
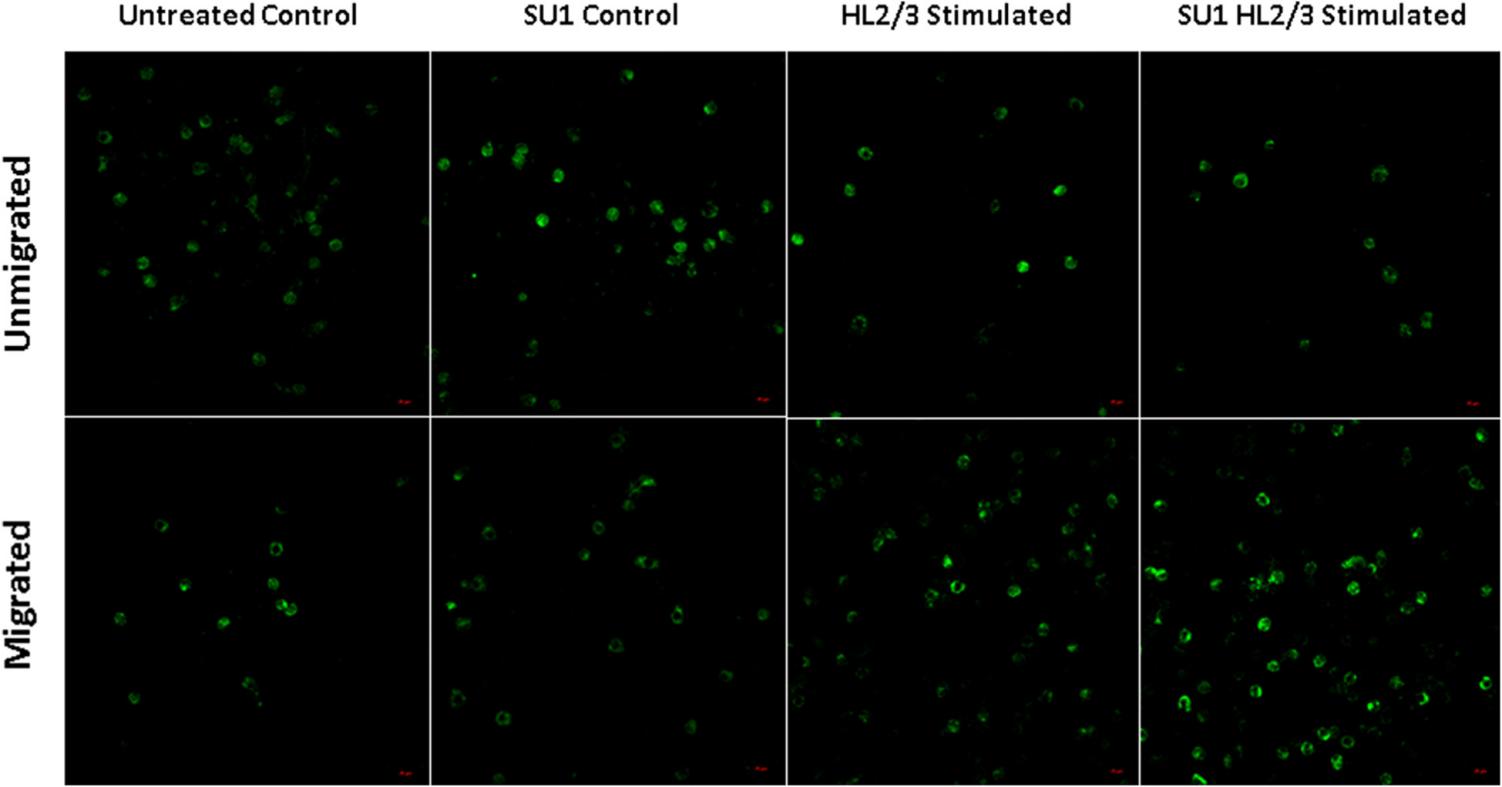
Representative images indicating the effect of *S.frutescens* extract on migration capacity of CD14+ (FITC) primary human monocytes across a simulated BBB. Magnification: 40× objective. SU1 indicates groups pre-treated for 4 h with *S. frutescens*

**Fig. 4:**
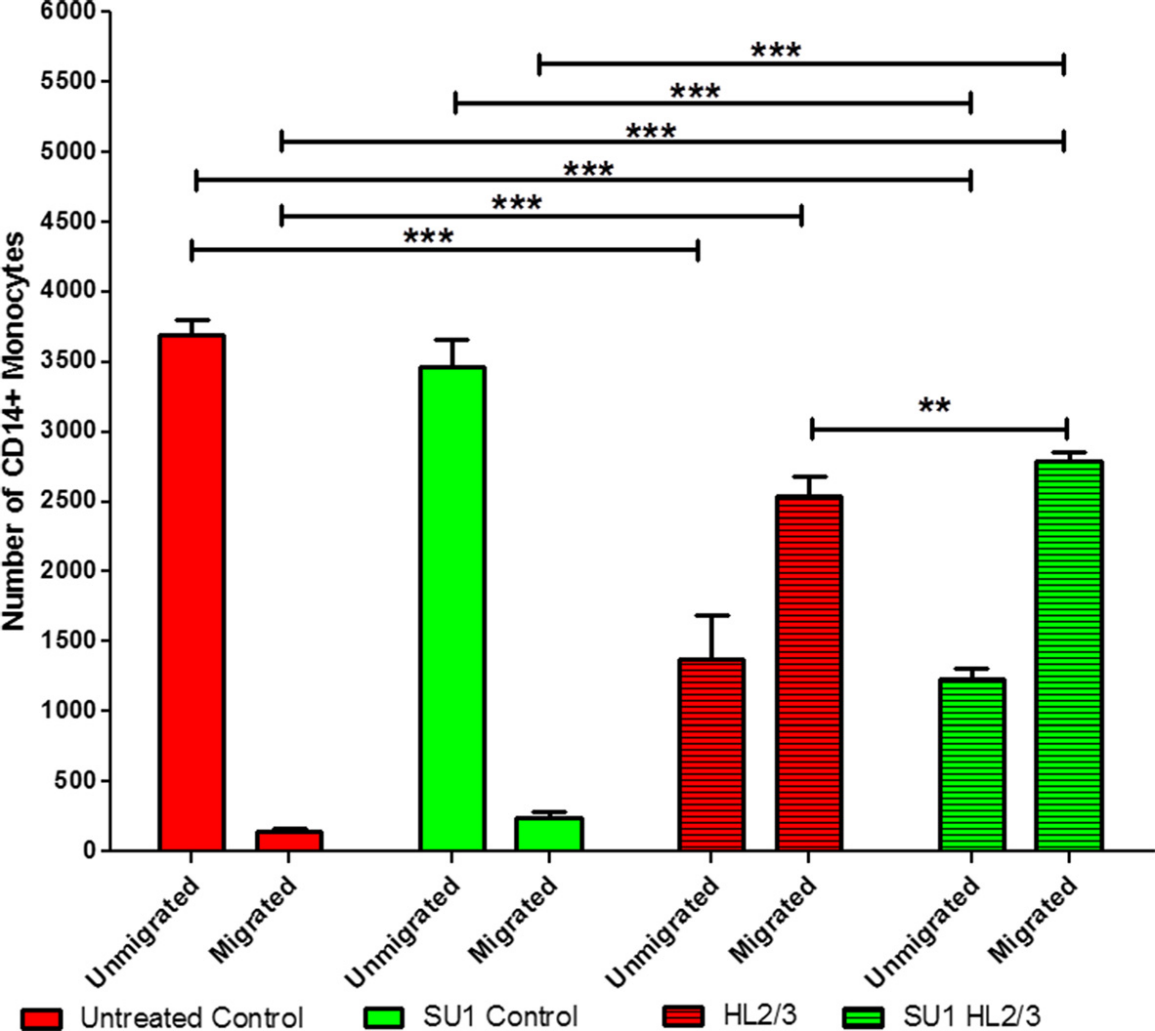
Effects of 4 h of *S.frutescens* extract pre-treatment on monocyte migration across an in vitro BBB. SU1 indicates groups pre-treated with *S. frutescens.* Results are expressed as mean ± SD. *** = *P* < 0.0001; ** = *P* < 0.001

Migration of primary human monocytes across the BBB was assessed, as well as the role of *S.frutescens* as modulator of this process. The number of monocytes remaining on top of the transwell filter insert (containing BBB) was named unmigrated cells, while those collecting in the bottom of the well are referred to as the migrated cells. Representative images of immunocytochemistry used to visualise monocytes for the purpose of quantification, are presented in Fig. 3 and illustrate the marked differences in CD14+ monocyte counts between the experimental groups. Numerical data are presented in Fig. 4. As anticipated, HL2/3 stimulation resulted in a significant increase in monocyte migration across the in vitro BBB (ANOVA main effect *P* < 0.0001). Pre-treatment with *S. frutescens* had no effect on migration in absence of HIV proteins, but exacerbated the monocyte migration capacity in response to HL2/3 stimulation significantly.

## Discussion

Neuroinflammation is central to the aetiology, progression and prognosis of neurocognitive disorders associated with HIV infection. In addressing this problem, it is of paramount importance to not only search for potential therapeutic modalities, but also to invest in development of the best investigative models with which to evaluate these potential therapies. We believe that our data, presented here, contribute significantly to the advancement on both these fronts. Firstly, a novel aspect of our study is the use of HL2/3 cells to simulate in a more physiologically relevant manner than other non-infectious methods commonly employed, conditions following HIV 1 subtype B infection. Furthermore, to our knowledge no other group has employed this model to test the efficacy of a complimentary medicine that is currently recommended for use in a HIV population at risk of neuroinflammation.

In terms of the model, HL2/3 cells are mostly used in research focused on studying viral fusion mechanisms [18]. Here, we have used this cell type in a novel application, utilising its high-level production of a variety of HIV-associated proteins, including Gag, Env, Tat, Rev and Nef, to stimulate neuroinflammation in vitro. The simulated BBB co-culture, originally used for testing chemokine and monocyte migration responses to HIV-1, [19] was used here in a broader application to investigate efficacy of a natural extract. Proof of the accuracy of the model is the fact that data generated from the HIV-1 subtype B & C Tat stimulation experiments – showing that subtype B is more inflammatory than subtype C – are congruent with previous reports in the literature [20]. The combined use of the BBB model and HL2/3 cells substantially improved the outcome of our investigation. For example, results suggesting that *S. frutescens* aggravates the MCP-1 response independent of Tat, may have been missed using stimulation with single proteins alone. We confidently recommend the use of this model to evaluate modulatory effects of various compounds/drugs/medicines which may have the potential to modulate HIV-1 induced inflammatory processes within and surrounding the neurovasculature.

**Fig. 5:**
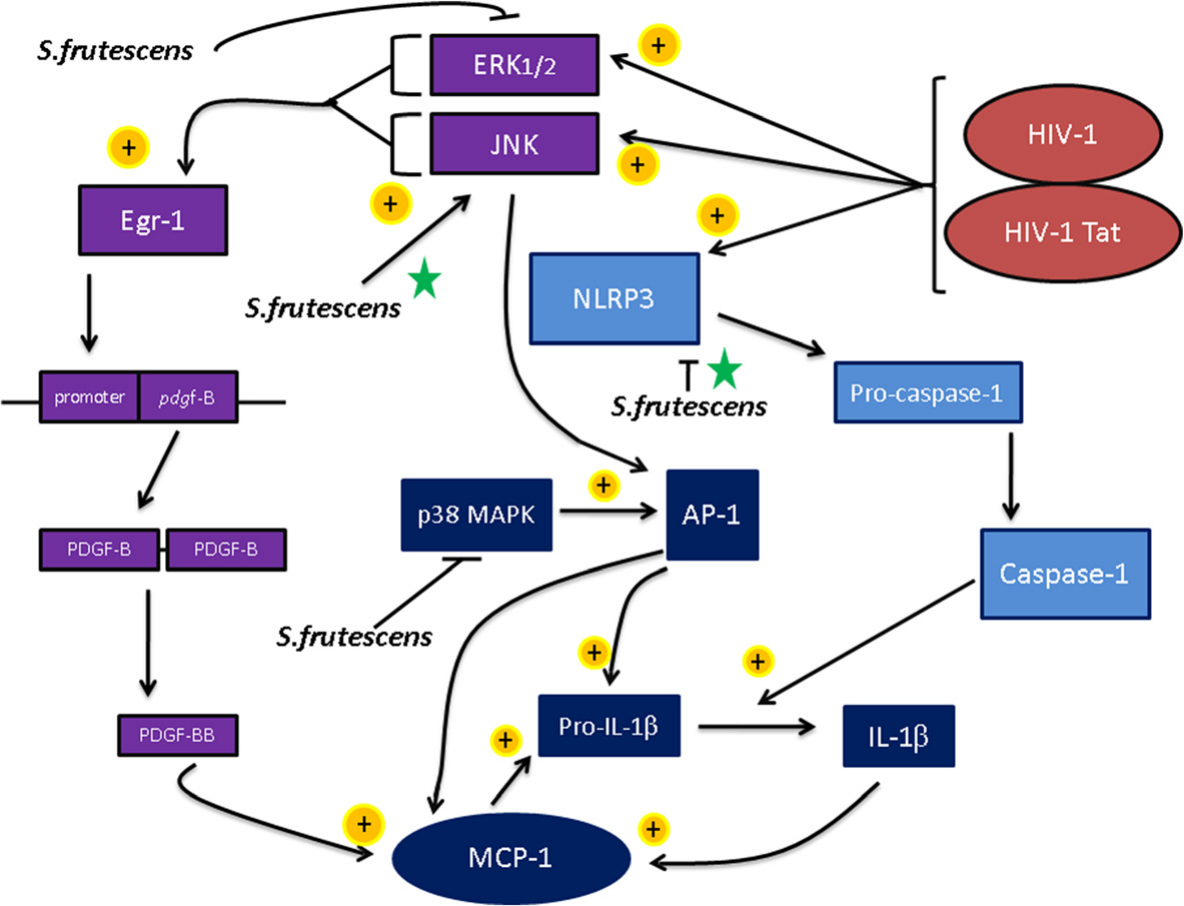
Schematic representation of inflammatory signalling pathways associated with HIV-1 infection. Novel mechanism of action elucidated by current data is indicated with a green star. Abbreviations: Mitogen activated protein kinase (MAPK); Extracellular signal-regulated kinase 1/2(ERK1/2); c-Jun N-terminal Kinase (JNK); Early Growth Response protein-1 (Egr-1); NLRP3 – NOD-like receptor family, pyrine domain containing 3

Turning our attention to the complementary medicine evaluated, at first glance results may seem contradictory, with both HIV protein-stimulated MCP-1 production and monocyte migration suggesting a pro-inflammatory effect of *S.frutescens*, while the decreased IL-1β levels after pre-treatment seems to argue against this interpretation. However, these results may be explained by delineating the signalling pathways involved in pro-inflammatory cytokine production, which are affected by the plant. A simplified signalling pathway is presented in Fig. 5, illustrating three main avenues by which the production of MCP-1 and IL-1β are affected.

HIV-1 and its associated proteins set off an inflammatory cascade, activating all three these signal transduction pathways, one via direct activation of the NLRP3 inflammasome, [21] and the other two by activation of either of ERK1/2 or JNK [22]. *S. frutescens* has been the subject of research for a number of years due to its claimed effectiveness against cancer, stress and cachexia – this research elucidated some signalling targets of the plant relevant to the current study. For example, *S. frutescens* has been reported to inhibit activation of ERK1/2 [23, 24] and p38 MAPK [6], albeit in non-HIV models. In the context of our data, inhibition of ERK1/2-activation of the Egr-1 and p38MAPK pathways did not eliminate signalling to achieve MCP-1 production, which was increased in the presence of HIV proteins, and even further after *S. frutescens* pre-treatment, suggesting a relative up-regulation of the activation of these pathways via JNK in a cumulative manner by HIV proteins and *S. frutescens*. This up-regulation of JNK after HIV infection has been reported previously in the context of HIV-associated neurocognitive disorders, where it was associated with up-regulation of AP-1-mediated increases in pro-inflammatory cytokines IL-6 and IL-8 [25]. However, our finding that *S. frutescens* exacerbated this neuroinflammatory response is novel.

Additionally, the phosphorylation of the transcription factor AP-1 is known to enhance its transcriptional control of genes involved in the inflammatory process, in this case MCP-1. The 5’-flanking region of the MCP-1 gene contains multiples AP-1 binding sites [26], thus increased flux through the JNK pathways would naturally lead to increased MCP-1 gene expression and consequent protein translation. The fact that this JNK-mediated up-regulated MCP-1 response did not translate to increased IL-1β levels – as in fact it did in the presence of HIV proteins – suggests that *S. frutescens* may also inhibit the NLRP3 inflammasome, which is the predominant pathway responsible for conversion of pro-IL-1β to IL-1β by caspase-1 (also called IL-1 converting enzyme) [27]. The latter may also explain the anti-inflammatory function of *S. frutescens* reported here under non-HIV basal conditions (when JNK is usually not activated and ERK activity predominates to deliver a flux of relatively smaller magnitude). This suggests that while *S. frutescens* may well have an anti-inflammatory effect which is potentially useful under basal conditions, the opposite is true in the presence of HIV proteins: under these conditions, JNK-activated pro-inflammatory pathways predominate and are further enhanced by *S. frutescens*. This effectively overcomes the previously reported *S. frutescens*-induced inhibition of similar pro-inflammatory pathways which do not predominate under these conditions, effectively resulting in a net pro-inflammatory outcome, as supported by our result of an increase of inflammatory leukocyte migration across the simulated BBB. This up-regulation of JNK in an HIV model suggests a new target for *S. frutescens* which has not yet been elucidated.

Extrapolating our data to a clinical application, CNS infiltration of both HIV-1 infected and uninfected monocytes is one of the main routes by which the virus enters and seeds the CNS as a viral reservoir to initiate neuroinflammatory processes. Thus, in order for any anti-inflammatory modality to be useful in this context, it would need to modulate this response – as we have previously demonstrated for grape seed-derived polyphenols [28] – which *S. frutescens* does not, and which it in fact seems to aggravate. Thus, in our opinion the use of *S. frutescens* should be avoided in the presence of HIV infection, as our data suggests that *S. frutescens* may increase monocyte infiltration into the CNS of HIV patients. Staying with clinical application, a recent study by Fasinu et al. [10] has also cautioned the use of *S. frutescens* by HIV+ patients on ARVs, due to the fact that the herb is able to inhibit enzymes involved in the metabolic clearance of these drugs. Given this data, the use of *S. frutescens* as complimentary medicine to patients already on ARV has been discouraged. However, use of medicinal plants by HIV-patients waiting for roll-out, is currently recommended by the Ministries of Health in several African countries – in South Africa, *S. frutescens* is recommended in this context [9] for prevention of cachexia, a beneficial effect of the plant previously reported by our group, as well as to prevent neuroinflammatory changes which were recently reported to occur in the early phases after infection, i.e., prior to ARV roll-out [1]. However, no data supports the latter application and our data now suggests that *S. frutescens* may in fact promote development of neurocognitive disorders by exacerbating inflammation and not inhibiting it in the context of neuroinflammation specifically. We urge policy makers to incorporate this evidence in their education of this at risk population as well as the health care practitioners providing primary care to them.

## Conclusions

In conclusion, current data illustrates that the combined use of HL2/3 cells and the simulated BBB presents an accurate, physiologically relevant in vitro model with which to study neuroinflammation in the context of HIV/AIDS. In addition, our results caution against the use of *S. frutescens* as anti-inflammatory modality at any stage post-HIV infection.

## Additional file

Additional file 1:
**Cell viability over time after exposure to** ***Sutherlandia frutescens*** **extract.** Human astrocyte (HA), human umbilical vein endothelial cell (HUVEC) and primary human monocyte cultures were exposed to *Sutherlandia frutescens* extract (50, 500 and 5000 ug/ml) for up to 24 h, before viability was assessed using the MTT assay. From these data, the 500 ug/ml dose of *S. frutescens* was chosen for all experimental work, as described also in the methods section.

## Abbreviations

AIDS: Acquired immune deficiency syndrome
AP-1: Activator protein 1
ARV: Anti-retroviral
BBB: Blood–brain barrier
CNS: Central nervous system
DMEM: Dulbecco’s modified eagle’s Medium
EGM: Endothelial growth medium
Egr-1: Early growth response protein −1
ERK1/2: Extracellular signal-regulated kinase 1/2
FCS: Fetal calf serum
HAND: HIV-associated neurocognitive disorders
HIV-1: Human Immunodeficiency virus type −1
HUVEC: Human umbilical vein-derived endothelial cells
IL-1β: Interleuken - 1β
JNK: c-Jun N-terminal kinase
MAPK: Mitogen Activated Protein Kinase
MCP-1: Monocyte chemo attractant protein - 1
NLRP3: NOD-like receptor family, pyrine domain containing 3
PBS: Phosphate buffered saline
