# Supplementary figures and images for: Sutherlandia frutescens may exacerbate HIV-associated neuroinflammation
Source: J Negat Results Biomed. 2015 Jul 18;14:14. doi: 10.1186/s12952-015-0031-y (PMC4506629; doi:10.1186/s12952-015-0031-y)

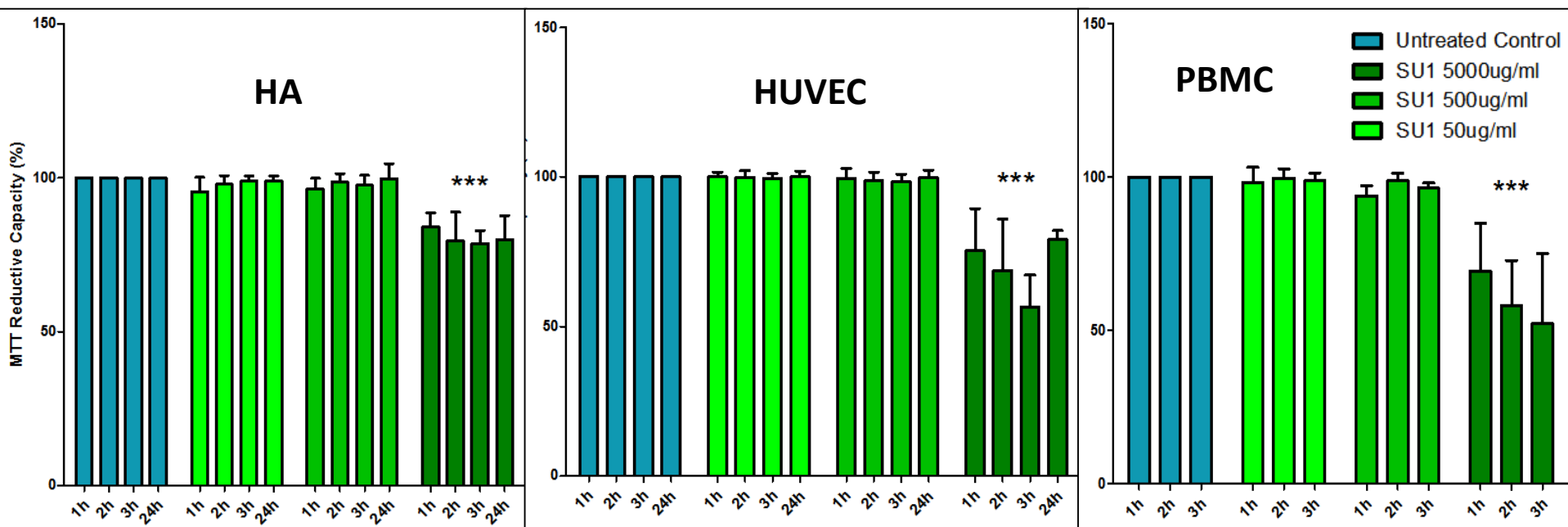

Supplement: Additional file 1: — Cell viability over time after exposure to Sutherlandia frutescens extract. Human astrocyte (HA), human umbilical vein endothelial cell (HUVEC) and primary human monocyte cultures were exposed to Sutherlandia frutescens extract (50, 500 and 5000 ug/ml) for up to 24 h, before viability was assessed using the MTT assay. From these data, the 500 ug/ml dose of S. frutescens was chosen for all experimental work, as described also in the methods section. [file 12952_2015_31_MOESM1_ESM.pdf]
